# Stigma and prejudice: the experience of crack users

**DOI:** 10.1590/1518-8345.0852.2680

**Published:** 2016-03-28

**Authors:** Nathália Duarte Bard, Beatriz Antunes, Cristine Moraes Roos, Agnes Olschowsky, Leandro Barbosa de Pinho

**Affiliations:** 1 Undergraduate student in Nursing, Escola de Enfermagem, Universidade Federal do Rio Grande do Sul, Porto Alegre, RS, Brazil. Scholarship holder from Conselho Nacional de Desenvolvimento Científico e Tecnológico (CNPq), Brazil; 2 Master's student, Escola de Enfermagem, Universidade Federal do Rio Grande do Sul, Porto Alegre, RS, Brazil; 3 PhD; 4 PhD, Full Professor, Escola de Enfermagem, Universidade Federal do Rio Grande do Sul, Porto Alegre, RS, Brazil; 5 PhD, Adjunct Professor, Escola de Enfermagem, Universidade Federal do Rio Grande do Sul, Porto Alegre, RS, Brazil

**Keywords:** Substance-Related Disorders, Qualitative Research, Health Evaluation, Mental Health, Social Vulnerability

## Abstract

**Objective:**

to evaluate the stigma and prejudice experienced by crack users in their social context.

**Method:**

a qualitative study developed through the Fourth Generation Evaluation, conducted with four interest groups (ten users, eleven families, eight employees, and seven managers), components of the mental health care network. For data collection, we used observation and individual interview. The analysis was performed through the constant comparative method.

**Results:**

crack users suffer prejudice and are stigmatized as those who do not fit in the systems established by society (without family links, formal employment and dwelling), and are thus excluded. They exhibit undisciplined behavior and, therefore, are discriminated, marginalized and considered as criminals, losing their uniqueness and living in vulnerable situations.

**Conclusion:**

the evaluation process emphasized the need to demystify the social imaginary that demonizes the chemically dependent, being thus important to develop public policies with actions focused on health, prevention, information and combat to stigma.

## Introduction

Currently, the abuse of psychoactive substances has also been addressed as a public health problem, and to deal with partnership with various sectors of society have been requiring, in a permanent exchange of ideas and information, due to the complexity of the situation and the rapid expansion of drug use. 

In this context, associated with the compromise of the social life of users and their families, the abuse of crack also triggers clinical compromises caused by the dependence of this substance and, in addition to the compulsive aspect of the drug, users generally get involved in fights and transgressions, and their daily lives are permeated by violence and crime^(^ 1 ^-^ 3 ^)^.

These issues caused by crack use end up leading to abandonment and loss of affective bonds, which causes social isolation and conflict with their support network. Such a situation has been on the media in the discussions of civil society and politicians, who have been emphasizing the negative aspects of drug addiction, and this has strengthened prejudice and stigma in relation to these users. The idea constructed in the social imaginary is that all users are involved with drug dealing, criminality and that quitting drugs is related to the user's will power. 

Thus, prejudice is understood as a premature and inadequate judgment about the use and abuse of drugs. That is, something or someone is defined based on an idea without prior knowledge. Prejudice is a negative judgment attributed to the characteristics of otherness; it implies the negation of someone who is different and, in this way, establishes one's identity as superior/dominant^(^ 4 ^)^.

On the other hand, stigma reveals something that extrapolates an attitude of prejudging, as something infamous, despicable and dishonorable, a stain on someone's reputation, and this infers contamination, infection, and transmission, making the isolation of the contaminant urgent and necessary^(^ 5 ^)^.

While the stranger is in front of us, we may see evidences that he/she has an attribute that makes him/her different from others, and even as a less desirable individual^(^ 6 ^)^.

Thus, based on prejudice, one does not consider the drug user a common and total human being, reducing him/her to a damaged and diminished person, i.e., stigmatizing the individual especially when no one believes him/her anymore. 

Prejudice and stigma related to/towards crack users have influenced the relationship of these people in various sectors of society, because they are related to criminality, and thus stigmatized, neglected and marginalized as citizens, which reinforces excluding and violent approaches.

We suggest that the therapeutic approach to users of psychoactive substances must be based on the particularities of each individual, considering aspects of consumption, vulnerability, risk and the increased access to and continuous care of the Brazilian Unified Health System (SUS)^(^ 7 ^)^.

Assuming this, we propose in this article to evaluate the stigma and prejudice experienced by crack users within their social context - a research funded by the National Counsel of Technological and Scientific Development (CNPq), called "ViaREDE - Avaliação qualitativa da rede de serviços de saúde mental para atendimento a usuários de crack (Qualitative evaluation of the network of mental health services for crack users)"^(^ 8 ^)^, whose goal was to assess the network of mental health services for crack users in a municipality of Porto Alegre, state of Rio Grande do Sul, Brazil. 

## Method

This is an evaluative and qualitative study that uses the Fourth Generation Evaluation methodological assumptions, characterized as a responsive and constructivist assessment in which the demands of interest groups are the focus of the assessment, built jointly in a hermeneutical dialectic process of interaction and negotiation between researcher and the interest group. Interest groups are formed by organizations, groups or individuals who have common interests in the evaluation process, and somehow are involved or are potentially affected by the issue^(^ 9 ^)^.

The research was conducted in a municipality of the metropolitan region of Porto Alegre, state of Rio Grande do Sul, Brazil. We collected the data through observation and previous ethnography, when the researcher interacted without being engaged in evaluation activities. The previous ethnography stage was registered in a field journal and took a total of 189 hours^(^ 8 ^)^. 

Regarding the interest groups, we can describe them as the following: *ten users* who attended the Psychosocial Care Center for Alcohol and Drugs (CAPS AD), or other mental health service network, and who were in good conditions of expressing themselves, being excluded those who were in a psychotic state, abstinence or had significant cognitive deficit; *eleven family members* that were participating in the follow-up provided by the CAPS AD during the data collection period; *eight employees* of the CAPS AD, with at least six months of experience in the institution, or in the mental health services network, who were not on leave or vacations during the collection: two psychologists, a psychiatrist, an occupational therapist, a workshop leader, a nurse, a nursing technician and an administrative assistant; and *seven professional managers*: five members of the Board of the Mental Health Institution (three psychologists, a social worker, an occupational therapist), a nurse coordinator of the mental health team of the general hospital and a nurse coordinator of the basic health care of the municipality^(^ 8 ^)^.

For the application of the data collected, we carried out: (1) contact with the field, in which the research proposal was presented and discussed, when the interest groups agreed to participate in the assessment; (2) organization of the evaluation process, when the researcher entered the project and made free observations, with the objective of knowing the reality and context of the service provided, without being engaged in the evaluation activities; (3) identification of the interest groups; (4) development and joint constructions, in which we held interviews through the dialectical hermeneutical circle; (5) expansion of joint constructions, in which extra information and materials that could contribute to the evaluation process were introduced; (6) establishment of the agenda to organize the information and the constructions from the groups that would be presented to the participants; (7) implementation of negotiation, in which respondents had access to the information obtained on data collection for the discussion, in a way they could modify the process, or to confirm its credibility reaching a possible consensus^(^ 9 ^)^.

The interviews were held with the application of the dialectal hermeneutical circle, in which the first respondent - named R1 - answers an open question about the object of evaluation: *talk about the health care provided to crack users in this municipality*. The respondent is asked to describe how he/she constructs the object of evaluation and to comment on it. The central topics, concepts, ideas, values, concerns and issues presented by R1 are analyzed by the researcher, who formulates the first construction, C1. Then, R2 is interviewed and, after commenting on the questions, the topics obtained by the analysis of R1 are introduced, and R2 is invited to comment on them. As a result, the interview of R2 generated information not only from R2, but also criticism on the construction made by R1. The researcher completes the second analysis, resulting in the C2 formulation, which is considered a more sophisticated construction, based on two sources of information: R1 and R2. This process is the beginning of the final construction and it is repeated with subsequent interviews until all the participants in the circle answer the question^(^ 9 ^)^.

The interviews were held individually, recorded and entirely transcribed - in this research, the letter P (participant) identifies the statements.

For data analysis, we used the Constant Comparative Method, and thus the analysis was performed concomitantly with data collection. This method has two steps: identification of information units and categorization. The information units are sentences or paragraphs obtained from the empirical material, registered to be understandable to any reader and not only to the researcher. The categorization aims to unify into temporary categories all information units related to the same content. We aimed, thus, at internal consistency in the categories to - after negotiation - establish definitive ones^(^ 10 ^)^.

The evaluation process emphasized the following thematic categories: network design, network management, network access, network articulation, media and crack, prejudice and stigma, prevention campaigns, features of the work in mental health care, user's characteristics, strategies of the work in mental health care, employee's profile, health care provider's background. In this paper, we discuss the information units of the thematic category "stigma and prejudice in relation to crack users".

The ethical principles were ensured according to Resolution No. 466/12 (BRAZIL, 2012), from the Brazilian National Health Council of the Ministry of Health^(^ 11 ^)^. The project was approved by the Ethics Committee of the Universidade Federal do Rio Grande do Sul (CONEP UFRGS), under No. 16,740. All participants signed the free and informed consent form.

## Results and discussion

In the evaluation process, topics such as prejudice and stigma related to crack users were pointed out. These users suffer the negative consequences of being labeled and stereotyped as undesirable and unproductive human beings, which put them in the lowest position in the social hierarchy and may interfere in relation to opportunities as citizens and in their lives in society. Thus, the loss of status itself becomes the basis of discrimination, stereotyping, and segregation^(^ 6 ^)^.

Relating the concepts of prejudice and stigma in relation to crack use, we could consider social hierarchy through the evaluation process. In this hierarchy, crack users are labeled and considered outsiders in the social context - those who do not fit in the systems established by society (no family links, formal employment and dwelling), including the idea that they should be excluded. They are seen as different and inferior people. According to the law, any citizen can enjoy the public spaces of our city; however, the society in which these people are included believes they do not have this right or that they cannot be considered citizens.

*The capitalist society establishes the licit and illicit consumption of goods and products. In relation to crack, the user frequently consumes it in public spaces, occupying these places. Most people believes in the social imaginary that these people must be removed from the street, in order to "clean" these spaces* - *an urban cleaning*(P1)*.*

Therefore, it is necessary to construct a society that does not moralize life situations or the problems faced by citizens, because these factors influence the right of using public spaces that belong to everyone.

Concerning the issue of crack and drug addiction, before moving, preventing access and hiding, society must demystify the idea that the user is someone incapable, dangerous and without conditions. We need to disseminate information and deal with this problem as a health issue. We also must include the economic, educational and social assistance sector, in addition to policies, proposing the right to health care, access to public spaces and, especially, to support healthcare institutions that are prepared to assist this kind of user. To deal with drugs we need to combat prejudice and stigma and, thereby, health assistance is oriented by the production of social life.

It is possible to affirm that prejudice and stigma are very similar social processes that can result in discrimination, involving categorization and labeling, stereotyping and social rejection^(^ 12 ^)^.

Among the held interviews, the statements of respondents show that drug users are judged and stigmatized in different ways, depending on the type of substance they use, even among them. Those who smoke crack suffer another type of discrimination, which is more intense.

Here in CAPS, when they present themselves as a group, the alcoholic says "I drink alcohol, only alcohol", that means "I'm better than those who smoke crack, because I just use alcohol" (P2).

This is not real; it hinders the work and separates people. "Oh, I am an alcoholic, you are a crack user". How can I group them, if there is prejudice among them? It's hard to deal with it sometimes. (P3).

This greater prejudice and stigmatization of crack users exceed territorial barriers. In a study conducted in New York (USA), drug users stated that powdered cocaine users are less likely to experience stigmatization and the subsequent negative treatment in comparison with crack users^(^ 13 ^)^.

The stigmatized person has two identities: the real and the virtual. The real identity is the set of categories and attributes that a person proves to have; and the virtual identity is the set of categories and attributes that people have when they show themselves to strangers, so these are demands and character features - made by those considered normal - in relation to who is the stranger^(^ 6 ^)^. In this way, the virtual image of crack users is recognized as a damaged identity that represents something bad within society and therefore they should be avoided.

It is noticeable that the virtual identity of the crack user is considered an indisciplined behavior. That is, it is clearly an invidious and discriminatory attitude that generates situations of vulnerability, in which crack users are seen as a social barrier. 

Society has diagnosed and generalized crack users as marginal subjects and criminals. However, it is known that this diagnosis creates an identification that gathers a group of individuals according to a certain meaning, abolishing their particularities, and can thus negatively affect the individual's life, since every diagnosis involves value judgment and, as a result, segregation^(^ 14 ^)^.

We must draw attention to the fact that the use of crack is not equivalent to delinquency and criminality. This idea is impregnated by an emotional climate derived from the stigma that discriminate and affects the life of users, families and society. We need to realize that dependence on a substance is not only conditioned by the person's will, because there are physiological and psychological needs involved in this situation. Addiction is not a matter of choice for users; they are hostages of the drug.

The instant euphoria that the drug brings reinforces and motivates individuals to use it repeatedly, establishing an intimate relationship between users and the drug. Facing everyday problems, chemically dependents find in drugs a way to overcome their frailty. Thus, it is an arduous task of elaborating and implementing effective measures to fight against this substance^(^ 15 ^)^.

Based on the considerations above, to see crack users without predetermined labels, without fear and without the idea it is a problem - or a danger - health professionals have the task of engaging in the challenge to change this picture. These professionals have to be committed and consider the real identity of crack users, something that points out to an approximation to the lives of these people and not just to the idea reported by the media, which enhances the virtual identity.

Through permanent education, it is something necessary and important for employees of the health care network. I think that we must deconstruct this image created by the media that portraits the crack user as a "zombie", as a person who is totally lost, the idea that if you become addicted there is no hope (P4).

In this sense, we must put aside prejudices and preconceptions, and "to do this, it is necessary a human assistance, characterized as a greater sensibility to listen without prejudices. Such assistance can be put in practice by changing attitudes, in search of new knowledge, improvement of skills and recognition of the other, from what is considered non-existent"^(^ 16 ^)^.

During the evaluation process, we found that crack users and society are within the same context, but in constant conflict. On one hand, the society that discriminates and, on the other, the user who does not want to be condemned or victimized - he/she just want to be considered a part of society, with challenges and frailty to be faced. The misjudge concerning crack users makes even more difficult their treatment and social reintegration.

All sectors of society are responsible for combating the prejudice related to the discrimination of crack users, which is a barrier that should be removed. The objective is to include the citizen that has problem with drugs, considering them people with rights and duties in the participation and accountability of their social life. The idea is to demystify.

I think it's a basic thing, but it's a plan, it's a cultural thing, and we need to change the way of thinking about crack. It's a pathology, an issue related to health, yes, it causes damage, but this must be named in these terms (P4).

I think the main factor is to know the users. Forget about prejudice, this idea that the crack user will steal or kill people. I think that not having prejudice is the best alternative, because otherwise what will happen to these people? (P5).

 There is a clear need to change the culture of exclusion. Although it is a slow and gradual process, it should be increasingly encouraged by the health and education sectors. Thus, "the deconstruction of the exclusion paradigm of the person who has a life of suffering and the construction of a new one bring the perspective of living with differences, it is a process"^(^ 16 ^)^.

 In other words, the issue of crack and other drugs show how much society needs to review its concepts about differences, to change its values, accepting the other as he/she is. This intolerance of society is increasingly evident in the case of the crack use. 

We know that there is a group. It is a group that involves politicians, doctors, anyway, a society that does not agree with the idea of taking care of people, something that has to do with prejudice, with our madness. The difference has to be put aside, the moral aesthetic standards need to follow a certain path, and I think that this group now find it in the AD issue and crack exposes this situation. The crack exposes this difference, the lack of access of people to several things, education, housing, salary, and part of society cannot deal with this, does not want to deal with it. So, the AD issue in crack reveals the grotesque inability to deal with madness (P6).

The repressive logic focus on the drug, what results in no definitive solutions regarding the attention to the individual. To believe in these subjects, it is necessary to listen to them and to accept the use of drugs regardless of the legal aspects that it involves. Working with public health and drugs means accepting that their use is a fact, and it does not necessarily leads to dependence, and there are different risks related to the use of drugs^(^ 17 ^)^. Generally, the marginalized minorities are those living in *favelas*, in addition to the drug dealers themselves - this population consists mainly of black people and migrants from the Northeast region of low-income or living in extreme poverty conditions^(^ 18 ^)^.

Prejudice and stigmatization in relation to chemically dependents often hides the real situation of vulnerability in which the user is living in. The stigma is identified in the production of violence. From the experience of these peripheral communities, it is clear how the lack of job opportunities and infrastructure become facilitators for the expansion of crack use, i.e., the situation of poverty and the State's flaws would stimulate the use of this drug^(^ 19 ^)^.

 Thus, during the evaluation process it is possible to perceive the need of investments to face the crack issue. To this end, it is essential to demystify the social imaginary that demonizes the chemically dependents, which consider them marginalized individuals and criminals. Now is the time to invest in the integration of public policies with health education, prevention, information and combating stigma so the treatment of chemically dependents can be positive and reintegrate them into society.

## Conclusion

The evaluation process showed that the society see the crack user based on the user's virtual image, disregarding his/her real image, i.e., it does not see him/her as a singular person, with a life history, feelings, desires, learning, gains and losses. A stigmatizing identity of marginals, bums and violent persons predominates, creating the idea of non-citizens, without a social place, the ones who should be excluded. 

With this, the construction of the crack user's life is driven by stigma and prejudice, which results in discrimination and resistance in living with these people, because they are often associated with irresponsible practices, unrestricted pleasure, delinquency and the confrontation of socially accepted habits and customs.

These biased information about the use of crack and the way users are treated generate discussions that can be strategic for the increase or decrease of stigma and discrimination. It is necessary to discuss the practices and knowledge, stating the actual concept of drug use, which does not mean delinquency and criminality, as opposed to emotional climate mobilized by this invidious conception that comprises the user's life and his family, who are seen as social outcasts. 

We live in a territory where multiple disputes, policies and actors exist, where everyone adopts values and conceptions established by moralists, and the stories of people's lives are disregarded and relegated to a second fiddle. To discuss prejudice against the use of drugs without moralizing it can result in a process of negotiation and accountability in society, reverting the situation into awareness regarding this topic. Such a question could result in greater access of the users to health care networks and to public spaces of the city, which shows the importance of discussing this subject in this research.

Generally, people do not listen to drug users because there is a "consensus" in society that result in "deafening" in relation to the possibility of listening to them and to an ethical and dignified reception. Therefore, we reaffirm the importance of a psychosocial care network to deal with the use of crack, i.e., health services and society. Actions are required in this territory, an extensive fighting against moralism and prohibition, because users and their families need attention and care.

In another context, public policies must also provide information to deal with and understand this reality, in which the user is a citizen of rights, in which critical reflection and educational acts are focused on alternative actions of prevention, rehabilitation and social reintegration. 

This also permits the share of decisions between individuals, so they can have control over the assessment project, appropriating the various evaluation steps and participate in society through the hermeneutical interpretation and dialectical understanding to deal with conflicts and generate consensus. The formative character of this process, the search for the classification of information and the empowerment of interest groups stand out as relevant contributions in the fourth generation evaluation, consisting of aspects related to its systematization. These, despite also being important in other approaches based on participatory evaluation, in some situations are not guaranteed in the practical application of these evaluations. 

We considered that this study, within a participatory evaluation process, gave voice to crack users, their family members, health care workers and managers, promoting reflections on prejudice and stigma, and opening space for discussion and changes needed to combat the discrimination that disregards the individual as a citizen. 

Regarding limitations, the methodology does not establish the evaluation focus*a priori*. Considering that there is no tradition of this type of discussion among drug users, families, health care managers and staffs, we debated the problems and difficulties in a collective way - and this may have been a obstacle for all data to be included in the negotiation meetings, with the problematization in light of the psychosocial paradigm. 

We considered that the issue of crack concerns everyone - all professionals, politicians, and citizens. Each one of us must act, according to our fields and areas, to ensure the rights of everyone and to treat and reinsert crack users into this society full of particularities.
